# Supplementary material for: Mutations in PCYT2 disrupt etherlipid biosynthesis and cause a complex hereditary spastic paraplegia
Source: Brain. 2019 Oct 22;142(11):3382–97. doi: 10.1093/brain/awz291 (PMC6821184; doi:10.1093/brain/awz291)
Supplement: awz291_Supplementary_Data [file awz291_supplementary_data.zip › awz291-Suppl_data/Supplementary_Data4.zip!/Supplementary information 3 - fibroblast lipidomics.zip!/Fibroblast lipidomics/Controle vs Patient2/Statistical plots and data/PE[O] species/Volcano plot_alternative.pdf]

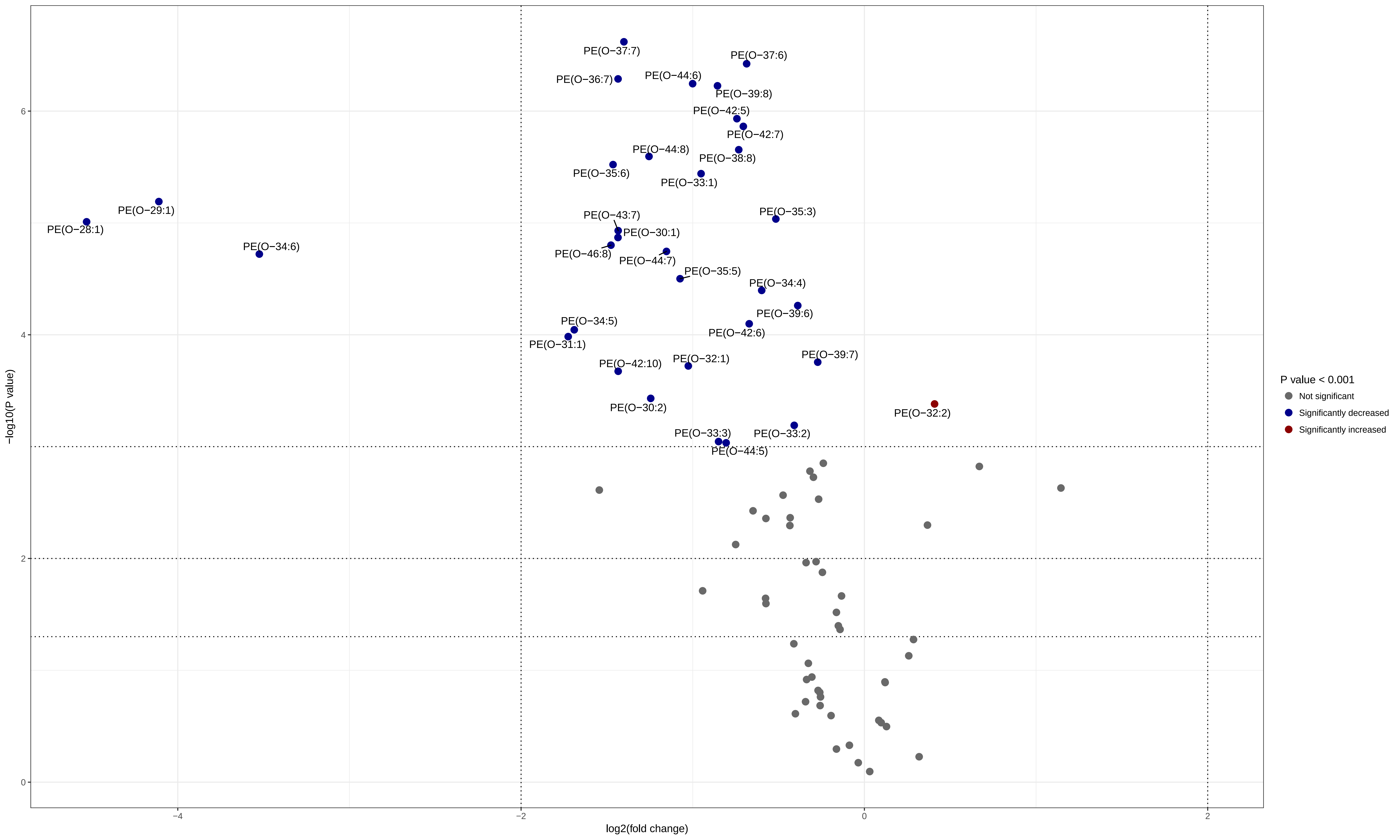

PE(O−37:7)
PE(O−37:6)
PE(O−36:7)
PE(O−44:6)
PE(O−39:8)
PE(O−42:5)
PE(O−42:7)
PE(O−44:8)
PE(O−38:8)
PE(O−35:6)
PE(O−33:1)
PE(O−29:1)
PE(O−28:1)
PE(O−43:7)
PE(O−35:3)
PE(O−30:1)
PE(O−34:6)
PE(O−46:8)
PE(O−44:7)
PE(O−35:5)
PE(O−34:4)
PE(O−39:6)
PE(O−34:5)
PE(O−42:6)
PE(O−31:1)
PE(O−39:7)
PE(O−32:1)
PE(O−42:10)
PE(O−30:2)
PE(O−32:2)
PE(O−33:3)
PE(O−33:2)
PE(O−44:5)
6
4
2
0
−4
−2
0
2
−log10(P value)
log2(fold change)
P value < 0.001
Not significant
Significantly decreased
Significantly increased
